# Editorial: Current perspectives on Developmental Coordination Disorder (DCD), volume II

**DOI:** 10.3389/fnhum.2024.1466104

**Published:** 2024-07-31

**Authors:** Kate Wilmut, Catherine Purcell, Emily J. Meachon

**Affiliations:** ^1^The Centre for Psychological Research, Oxford Brookes University, Oxford, United Kingdom; ^2^School of Healthcare Sciences, Cardiff University, Cardiff, United Kingdom; ^3^Faculty of Psychology, University of Basel, Basel, Switzerland

**Keywords:** Developmental Coordination Disorder, co-occurrence, Attention Deficit and Hyperactivity Disorder (ADHD), social-cultural context, muscular control

## Introduction

Developmental Coordination Disorder is characterized by a difficulty with motor control and coordination which falls substantially below the level expected given an individual's age and opportunity for learning (American Psychiatric Association, 2022). Individuals with DCD also experience associated secondary consequences which include poorer mental health outcomes (Kirby et al., 2013; Draghi et al., 2020), physical inactivity (as described in this Research Topic by Purcell et al.) and challenges with executive function (Purcell et al., 2015; Sartori et al., 2020; Meachon et al., 2022). Despite a prevalence rate of ~5% (Blank et al., 2019), which is significantly higher than for autism, DCD remains under-represented within the academic literature and is often misunderstood among professionals (Meachon et al., 2024). This Research Topic aimed to capture current research focusing on DCD and this editorial draws out three themes from the collection of 10 articles.

## The social-cultural context

One consideration which is so often ignored within research is the environmental context of the participants, the society in which they exist and how the expectations of that society might change or influence behavior. Interestingly two papers within this Research Topic shed light on this environmental or social-cultural context. Abdollahipour et al. considered this within the context of their findings regarding reaching behavior, stating that the different environmental contexts and engagement in outdoor activities of the participants in their study in comparison to previous studies might explain some of the differences in findings. Furthermore, Kim et al. also considered how distinctive cultural factors in the Republic of Korea might influence the presentation of DCD, especially given the high rates of physical activity within that population. The consideration of DCD within different groups and populations is very important, especially in light of multiple studies drawing on the same sample, as identified by Purcell et al. within the physical activity literature in DCD. This, along with the dominance of western research may somewhat bias our understanding of DCD to one specific social-cultural context.

## DCD and ADHD

Children with DCD are more likely than children without DCD to have attentional difficulties, in fact it is estimated that 50% of children with DCD have co-occurring ADHD (Fliers et al., 2010). This is represented within this Research Topic with four of the articles making specific reference to this. Three of these considered the biological or neural underpinnings of DCD with an aim to make comparisons between children with DCD only and children with DCD and ADHD. Unfortunately, two of these articles which focused on the structure of gray matter (Malik M. et al.) and changes in gray matter following intervention (Malik M. A. et al.) didn't have a sufficient sample size to compare these groups but instead combined them into one to compare to typically developing individuals. Therefore, although these studies do not help us to understand DCD as separate to ADHD, they do provide important findings with regards to brain structure and re-organization after intervention. In contrast, a third study did make this comparison in adults and included a DCD only sample, an ADHD only sample, a sample with DCD and ADHD and a sample with neither. Meachon et al. used EEG to consider resting state neural differences across these groups. The identification of neural structures or mechanisms which are specific to DCD is vital to further our understanding of this as being separate to ADHD.

A final study considered DCD and ADHD in much more of an activity of daily living. Falemban et al. used a qualitative method to delve into the experiences of parents when crossing the road with their child. This consideration of the lived experience is so incredibly important in order for researchers to fully understand DCD and its co-occurrences.

## Considerations of muscular control

Finally, three studies included measures or proxies of muscular control. Two of these focused-on children with DCD and found that children with DCD have unique motor strategies in muscular activity during the experience of perturbations while standing (Harkness-Armstrong et al.) and EMG firing rate during a gripping task (Esselaar et al.). These studies demonstrate the importance of considering task complexity and variance of performance rather than just an overall average when describing movement control in children with DCD. Both of these studies represent research which is aiming to explain or describe behavior in children with DCD such as falling or bumping into objects and dropping objects while carrying. In the third study, Sumner and Hill considered oculomotor control in adults with DCD as an extension to a previous study in children. Despite the wealth of research which focuses on children (very often young children) with DCD, it is important to remember that DCD is a life long condition.

## Conclusions

Although small, this Research Topic represents the diverse research which is being undertaken in the field of DCD. Looking across these studies we can see the pertinent issues within the field and also the exciting progress in terms of understanding the underlying biology which is driving the behavior with which we are all so familiar, it also acknowledges the importance of considered DCD within the context of co-occurrence and differing levels of task complexity. However, this is also complemented by research which is considering the voice of individuals with lived experience and research which acknowledges the importance of environment or social-cultural contexts of behavior. This represents a new tone in current DCD research compared to the mechanistic work which dominated the field 20 years ago. As this research field grows it is important to identify differences in findings and put effort into explaining why these might occur. It is only by carefully considering all the underlying and contextual factors that might influence an individual that we can fully understand the behavior that we are attempting to describe. Regardless of the research methods or the task being used the shift in focus from describing a disorder to considering the person is such an important one to ensure research is serving the people on which it focuses.

## Author contributions

KW: Writing – original draft, Writing – review & editing. CP: Writing – original draft, Writing – review & editing. EM: Writing – original draft, Writing – review & editing.
